# Short-term impact of celebrating the international clinical trial day: experience from Ethiopia

**DOI:** 10.1186/s13063-017-2081-6

**Published:** 2017-07-17

**Authors:** Abebaw Fekadu, Asrat Hailu, Eyasu Makonnen, Anteneh Belete, Getnet Yimer

**Affiliations:** 1 Center for Innovative Drug Development and Therapeutic Trials for Africa (CDT-Africa), Addis Ababa University, PO Box 9086, Addis Ababa, Ethiopia; 2 Department of Psychiatry, School of Medicine, College of Health Sciences, Addis Ababa University, Addis Ababa, Ethiopia; 3 Department of Psychological Medicine, King’s College London, Institute of Psychiatry, Psychology and Neuroscience, Centre for Affective Disorders, London, UK; 4 Department of Microbiology, School of Medicine, College of Health Sciences, Addis Ababa University, Addis Ababa, Ethiopia; 5 Department of Pharmacology, School of Medicine, College of Health Sciences, Addis Ababa University, Addis Ababa, Ethiopia; 6 Department of Pharmaceutics, School of Pharmacy, College of Health Sciences, Addis Ababa University, Addis Ababa, Ethiopia

**Keywords:** Clinical Trials, Clinical Trial Index, International Clinical Trial Day, Low and middle income country

## Abstract

Just over 2 years ago on 20 May 2014, we celebrated the first International Clinical Trial Day (ICTD) in Ethiopia at the College of Health Sciences, Addis Ababa University. The main aim of the celebration was to express solidarity with clinical researchers, particularly clinical trialists, across the world. Since this first celebration, several major steps have been taken with potential for improving the conduct of clinical trials in our institution and more broadly within the country. These have included policy impact, particularly commitment from the government and the institution for supporting clinical trials and for the broader improvement of access to medicines. A Clinical Trial Unit, led by a multi-disciplinary team of researchers, has been established. A regional centre of excellence is being established to build regional capacity for translational research and clinical trials. These are important outputs attributable to the celebration of the ICTD. We encourage ICTD celebration at institutions conducting clinical research. This is likely to have unanticipated positive institutional, national and even regional consequences.

## Background

The global disparity in the conduct of clinical trials remains very high. Represented in terms of the Clinical Trial Index (CTI) [1], a simple measure of disparity in clinical trials and health service provision in general, the proportion of trials registered from Africa, for example in ClinicalTrials.gov, remains very low, a mere 2.4%. This is much lower, 0.7%, if one considers sub-Saharan Africa, excluding South Africa. Celebration of the International Clinical Trial Day (ICTD) annually may have some impact on clinical trials and clinical research in low and middle-income countries (LMIC). In this commentary, we describe the potential impact of ICTD celebration based on our experience of celebrating the ICTD in the past 2 years.

## Main text

The first celebration of ICTD in Ethiopia, at the College of Health Sciences, Addis Ababa University, took place just over 2 years ago on 20 May 2014 [1]. The celebration was mainly to express solidarity with clinical trialists. Since then, we have celebrated the event two more times (2015 and 2016). We report here what we believe were the major impacts of celebrating the ICTD both for our University, the country and perhaps for the region. In the spirit of solidarity with the pioneer of clinical trials, and as suggested elsewhere, we present these impacts under six headings [2].

1. Networking: the celebration provided opportunity for networking among clinical researchers and between researchers and regulatory bodies, including ethics committees. For example, relations with research institutions such as the Ethiopian Public Health Institute and the Armauer Hansen Research Institute and the Federal Ministry of Health have been strengthened.
2. Sharing of information: the meetings have encouraged sharing of information, specifically around the research engagement of clinical researchers through scientific presentations, mutual interactions between clinical scientists and joint applications for grants. The ICTD created the ongoing forum for such engagement.
3. Collaboration with national research organisations and industry: since the first celebration, we have received more attention from industry and have also been able to engage with a few companies interested in manufacturing medicinal products.
4. Impact on policy: policy engagement of the government in clinical research has improved. In our latest ICTD meeting, a vice minister for Science and Technology attended the meeting. The government has committed to improving access to medications. The university has committed to make clinical trials one of its priority research areas.
5. Clinical Trial Unit: a multidisciplinary clinical trial unit was established at the College of Health Sciences of the university after the first celebration. The clinical trial unit has established two clinical trial sites, including a phase-1 trial site.
6. Establishment of a regional centre of excellence: one of the major achievements, led by the clinical trial unit, has been the establishment of a regional Centre of Excellence for therapeutic innovations. The centre, called the Centre for Innovative Drug Development and Therapeutic Trials (CDT-Africa) [3] is funded by the World Bank after a thoroughly competitive bid process. CDT-Africa is located within Addis Ababa University and will develop into a regional hub for capacity development in translational research and clinical trials. Currently, CDT-Africa has partnered with five universities from Ethiopia and five universities from the Eastern and Southern Africa region. Several other international universities and research institutes have also partnered with the centre.

## Discussion

Our observation has been that celebration of the ICTD has triggered important changes within our institution and, to some extent, within the country, that enhanced clinical research and advocacy for improvement of access to treatment. The celebration has also strengthened collaboration among researchers conducting clinical trials, both nationally and internationally. These positive changes have the potential to influence broader research engagement. The establishment of a regional centre of excellence (CDT-Africa) will support capacity development and may attract talent for clinical research. This may also support regional influence. However, within the region, more needs to be done to attract and retain talent. Institutions need to be competitive in terms of institutional excellence and other incentives. The clinical research environment needs to transform drastically. Improving the research administration system and offering protected time to clinical researchers and strengthening research infrastructure are important. The growing emphasis on soft capacity (human capacity) is not sufficient. This should be supported by focus on infrastructure capacity. Ongoing leadership commitment and involvement of key stakeholders, particularly societal representation needs to improve. Participation of society can be enhanced through public advisory boards and participation of public representatives in ethics committees. Celebration of the ICTD will continue to advocate these changes. The celebration will also continue to provide a forum for knowledge exchange, networking among clinical researchers and engagement between researchers, research users and policy makers. We encourage other institutions to consider celebrating the ICTD.

## Conclusion

We have attempted to demonstrate the broader impact of celebrating the ICTD in Ethiopia. We think that this may be an important example for clinical trialists working or planning to work in LMICs. However, broader leadership support and commitment is essential to develop successful clinical research programmes in LMICs.

## Abbreviations

CDT-Africa: Center for Innovative Drug Development and Therapeutic Trials for Africa
CTI: Clinical Trial Index
ICTD: International Clinical Trial Day
LMIC: Low and middle income countries
